# Effects of Immersive Virtual Therapy as a Method Supporting the Psychological and Physical Well-Being of Women with a Breast Cancer Diagnosis: A Randomized Controlled Trial

**DOI:** 10.3390/curroncol31100477

**Published:** 2024-10-21

**Authors:** Oliver Czech, Aleksandra Kowaluk, Tomasz Ściepuro, Katarzyna Siewierska, Jakub Skórniak, Rafał Matkowski, Iwona Malicka

**Affiliations:** 1 Department of Physiotherapy, Wroclaw University of Health and Sport Sciences, 51-612 Wroclaw, Poland; 2 Supraregional Center of Paediatric Oncology “Cape of Hope”, Wroclaw University Clinical Hospital, 50-556 Wroclaw, Poland; 3 Lower Silesian Oncology, Pulmonology and Hematology Center, 53-413 Wroclaw, Poland; 4 Department of Oncology, Faculty of Medicine, Wroclaw Medical University, 50-367 Wroclaw, Poland

**Keywords:** breast cancer, physical activity, physical health, mental health, quality of sleep, virtual reality

## Abstract

This study aimed to evaluate the effectiveness of virtual reality (VR) in the mental state and quality of sleep improvement and physical activity (PA) increase of patients diagnosed with breast cancer (BC). A total of 33 subjects divided into experimental (EG, *n* = 17) and control (CG, *n* = 16) groups were assessed with the Mental Adjustment to Cancer Scale (Mini-MAC), International Physical Activity Questionnaire (IPAQ), Pittsburgh Sleep Quality Index (PSQI), and the Modified Hospital Anxiety and Depression Scale (HADS-M) at four time points. The experimental intervention consisted of eight VR TierOne sessions. Significant differences favoring the EG were identified in the group x time interactions for the main outcomes: destructive style of coping with the disease (*p* < 0.001), walking (*p* = 0.04), moderate (*p* < 0.001) and overall activity (*p* = 0.004), quality of sleep (*p* < 0.001), depressive symptoms (*p* < 0.001), anxiety levels (*p* < 0.001), aggression levels (*p* = 0.002), and overall HADS (*p* < 0.001). Trends, favoring the EG, in the constructive style of coping, sedentary behavior and intensive PA, and sleep efficiency and sleeping time were also found. A VR intervention improves general well-being in terms of the measured parameters.

## 1. Introduction

The most common cancer in women is breast cancer (BC) [1]. The diagnosis of BC is proven to be a mentally taxing event, and coping with the disease is very challenging [2]. Levels of psychical distress are often very high immediately after the diagnosis [3,4].

It has been proven that the diagnosis itself is a devastating experience. Available research confirms that a deterioration in psychophysical well-being not only occurs after the diagnosis of BC [5,6], but it is a very common phenomenon in the first moments after receiving information about poor health. According to a recent comprehensive study, the diagnosis itself is the main factor causing anxiety, depression, poor quality of sleep, and lower physical activity (PA) levels [7,8,9]. The variety of symptoms and threats means that the standards of oncological care, from diagnosis to the end of the treatment process, need a holistic and interdisciplinary approach, allowing it to cover a much broader symptomatology than the malignancy itself [10,11].

Fortunately, many factors help to improve the health and quality of life of patients at the stage of diagnosis [12,13]. The standard-of-care procedures usually provide access to psychological support at the diagnosis time point. Mostly, musicotherapy, aromatherapy, or relaxation techniques are used as standard procedures [14]. Also, the family plays an important role in improving quality of life through social activity. Krok et al. showed that marital status can affect the ability to cope with the disease [15].

Another important factor in BC pre- and rehabilitation is PA. The benefits of exercise for the BC patient’s quality of life, anxiety, and depression levels are described by Sun et al. [16]. According to his study, kinesiotherapy was an effective intervention for the improvement of quality of life and anxiety and depression symptom reduction. Scientists agree that it is not only the amount of PA that matters but also its intensity.

The multitude and diversity of symptoms and ailments, as well as factors influencing changes in the quality of life and general well-being of the patient, mean that new interventions can influence one’s health on many levels, and various aspects of physical health, mental health, and well-being are constantly being considered. Consequently, the last decade has seen an increase in the number of studies based on the use of virtual reality (VR) as a treatment method. The technological revolution has contributed to the implementation of modern equipment, including VR, in oncological treatment, and pre- and rehabilitation processes. Several studies have investigated and found a positive impact of VR on patient anxiety levels [17], post-traumatic stress disorder treatments, paranoid delusions and depression symptoms [18,19], and the discomfort management of chemotherapy treatments [20,21], as well as pain management during standard burn wound care procedures [22]. All the above benefits of the use of VR appear to be needed in the process of BC prehabilitation. Unfortunately, few studies have analyzed the durability of these therapeutic effects and their changes over time. Immersive technologies are known to have the ability to affect patient consciousness, which, combined with Ericksonian psychotherapy, creates a tool with high potential in mentally high burdensome diseases, e.g., cancer. Therefore, VR seems to be a promising intervention for oncological prehabilitation quality improvements, which results in better psychophysical conditions during treatment. According to recent studies, VR allows the patient to receive impressive therapeutical effects, with no or a low risk of side effects of the treatment, compared to other standard therapeutical methods such as musicotherapy, relaxation, or farmacological treatment [14,15,16,17,18,19,20,21,22]. Nevertheless, more extensive studies are needed in this area.

Therefore, this study aimed to assess the effectiveness of VR interventions on BC-diagnosed patient anxiety and depression levels, styles of coping with the disease, PA levels, and general quality of sleep during and post-VR treatment, as well as to determine whether the therapeutical effect will last the minimum eight weeks after the intervention has ended. The research hypotheses were as follows: H1—VR intervention improves the patient’s quality of life and general well-being, lowers depression and anxiety symptoms, improves the constructive style of coping with the disease and lowers the destructive style of coping, motivates them to higher and more intensive PA, and improves their quality of sleep compared to patients in the control group (CG) without the VR intervention. H2—participant improvements in quality of life and general well-being will remain at a similar level or improve within two months of completing the intervention despite undergoing oncological treatment, compared to a control group with standard-of-care procedures without additional interventions.

## 2. Materials and Methods

The research was conducted in June 2021 to October 2023 in the Breast Unit of the Lower Silesian Oncology, Pulmonology, and Hematology Center in Wroclaw, Poland. Patients were recruited for the study after being diagnosed with BC prior to a multidisciplinary team meeting to determine and present the patient with a treatment strategy. Inclusion criteria included patients who had not started treatment for breast cancer, regardless of the stage of the disease and the patient’s age. Patients with cognitive impairments described in the medical data file and those who had received psychiatric treatment in the past or during the study were excluded. All participants provided written consent to take part in the project. The study received approval from the Ethical Committee of the University School of Physical Education in Wroclaw, Poland (number 18/2019) and the approval of the Institutional Review Board of the Lower Silesian Center of Oncology, Pulmonology, and Hematology in Wroclaw, Poland. The study has been registered in the anzctr.org.au repository (ACTRN12623000085673).

**Figure 1:**
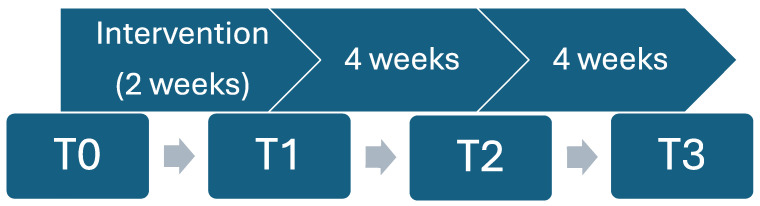
Graphical presentation of measurement time points.

Participants were divided into two groups: an experimental group (EG, VR group, *n* = 17) and a passive CG (*n* = 16). Randomization was based on a computer-generated list, according to the inclusion and exclusion criteria. The outcome measures were repeated across four time points: baseline pre-intervention (T0), post-intervention (T1—2 weeks past baseline measurement), follow-up measure (T2—6 weeks past baseline measurement), and 2nd follow-up (T3—10 weeks past baseline measurement). The intervention timeline is graphically presented in Figure 1.

The EG received 2 weeks of Virtual Therapeutic Garden (VR TierOne) sessions. The sessions were monitored by medical staff, additionally trained by the VR TierOne inventor and producer. Each session duration was about 15 min (min 13:50–max 17:50 for therapeutic sessions). Each EG patient had a total of 8 therapeutic sessions, with 1 session for demonstration and 1 session for summary. The sessions transferred patients to a virtual garden with therapeutic audio stimuli. The therapeutic sessions differed graphically. The condition of the virtual garden improved from session to session, starting with a weakened, colorless garden, leading to regaining colors, energy, and beauty at the last session. In addition, the audio commentary, which was designed as a comprehensive therapeutic cycle, could vary between sessions. In addition, all sessions included motor tasks (creating a virtual mandala, different for each session; tending the garden; and breathing exercises). The VR gear consisted of VR goggles and a controller (manipulator) connected to a PC. The use of a head-mounted display and the total immersion provides intense visual, auditory, and kinesthetic stimuli. The sessions were programmed to be calming and mood-improving. According to previous research on VR TierOne, the device helps patients recognize their psychological resources and motivates them to participate more actively in the rehabilitation process. In the virtual garden, the symbols and metaphors used are in line with the Ericksonian psychotherapeutic approach. The most important is the Garden of Revival, which symbolizes the patient’s health. Due the patient being involved in the garden’s cultivation, the virtual land begins to buzz with life, symbolizing the patients’ healing process. The power of metaphorical communication lies in bypassing patient resistance, as during therapy the patient’s state of health or life situation is not mentioned directly, but it shows an analogous process that unfolds before the eyes of the user. The inspiration of the Ericksonian approach allows the patient to undergo self-repair processes, which extend the therapeutic effects [23,24,25].

All participants completed a sociodemographic questionnaire with basic personal information and data regarding education level, residential and financial status (4-point Likert scale), and marital status. Additionally, we retrieved medical information such as estrogen and progesterone receptor expression, human epidermal growth factor receptor 2 (HER2)-positive BC, a Ki-67 proliferation index > 25%, and clinical tumor node metastasis (cTNM) cancer staging. The participants completed 4 standardized questionnaires concerning anxiety and depression levels, style of coping with the disease, PA assessment, and evaluation of quality of sleep.

The Mental Adjustment to Cancer Scale (Mini-MAC) was used to assess the participant’s response to the cancer diagnosis. This 29-item questionnaire is a widely used tool to assess coping strategies among cancer patients, based on a 4-point Likert scale. The questionnaire assesses strategies used to cope with cancer. The results range from 7 to 28 points. The scores can be divided into four categories: anxious preoccupation, fighting spirit, helplessness–hopelessness, and positive redefinition. Anxious preoccupation and helplessness–hopelessness points are summed to achieve the destructive style of coping score, and fighting spirit and positive redefinition are components of the constructive style of coping [26].

PA levels were measured using the International Physical Activity Questionnaire (IPAQ). The scale allows the calculation of PA and sedentary behaviors in adults and is based on 7 questions regarding activities over the previous 7 days, according to 5 domains: (1) occupational PA; (2) transportation PA; (3) housework, house maintenance, and caring for the family; (4) recreation, sport, and leisure time; and (5) time spent sitting. Each domain allows the assessment of the average quantity, duration, and intensity of PA. In this questionnaire, PA is expressed in metabolic equivalent (MET)-min/week units, which allows one to classify respondents into 1 of 3 categories of activity: insufficient (less than 600), sufficient (600–1500 or 600–3000), and high (more than 1500 or 3000 MET-min/week) [27].

The Pittsburgh Sleep Quality Index (PSQI) allowed the assessment of the quality of sleep. The PSQI is a 19-item scale for measuring quality of sleep and sleep patterns. This scale is widely used both in everyday practice and in clinical research, due to its high reliability. It assesses the quality of sleep according to 7 categories: (1) subjective QOS, (2) sleep latency, (3) sleep duration, (4) habitual sleep efficiency, (5) sleep disturbances, (6) use of sleeping medication, and (7) daytime dysfunction. The items are scored on a 4-point scale, and the global index score is calculated by summing the 7 category scores to provide an overall score ranging from 0 to 21, where a higher score denotes a lower quality of sleep. The questionnaire covers the month prior to the examination [28].

The Hospital Anxiety and Depression Scale Modified Version (HADS-M) is used to assess anxiety and depression in hospitalized patients. The questionnaire contains two independent subscales used to assess anxiety and depression; each subscale consists of seven statements. The questionnaire was enriched with two statements regarding the level of aggression. Answers are given on a 4-point Likert scale (0–3). The final score for each subscale ranges from 0 to 21 points. For the two questions regarding aggression, the scores range from 0 to 6 points. Results in the range of 0–7 (0–2 for aggression) indicate a normal result, a score of 8–10 points (3–4 for aggression) indicates a borderline level, while the range of 11–21 (5–6 for aggression) is considered a significant symptom [29].

All statistical analyses were conducted using JASP version 0.18.1 (University of Amsterdam, the Netherlands). A distribution analysis was performed using the Shapiro–Wilk test. Baseline data between the groups were compared using the unpaired *t*-test for continuous variables and the Chi-squared test for categorical variables. The impact of the intervention at specific time points was calculated using a repeated measures analysis of variance (ANOVA). The Scheffe test was used for post hoc analysis. In all analyses, the statistical significance was established at *p* < 0.05. The analysis of variance was extended using a linear mixed model (LMM), with group (EG and CG) and time (T0 to T3) as fixed effects.

## 3. Results

The participants included 33 women, 17 in the EG and 16 in the CG, who had been diagnosed with BC. Estrogen receptor expression was found in over 76% of patients in the study group and 100% of the CG. Progesterone receptor expression was seen in almost 65% of participants in the study group and all participants in the CG. HER2-positive BC was found in more than 70% of study group patients and 69% of CG patients. A Ki-67 proliferation index > 25% was observed in 59% of the study group population and 31% of the CG population. The quantities of patient cancer staging according to the cTNM cancer staging for each group are presented in Table 1.

The baseline demographic characteristics of participants included in this study are presented in Table 2.

**Figure 2:**
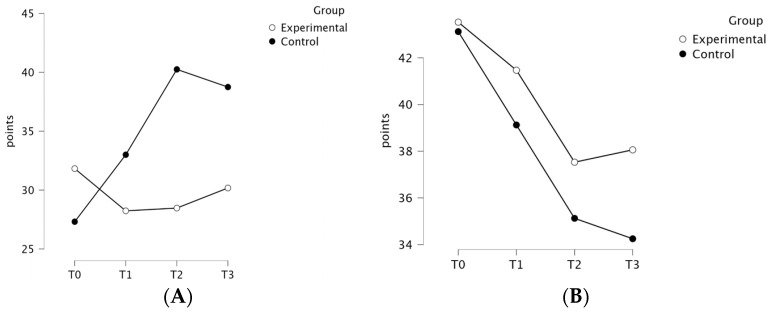
Comparison of destructive (**A**) and constructive (**B**) styles of coping (Mini-MAC) outcomes for the experimental and control groups at all measured time points.

The ANOVA revealed a significant group × time interaction for a destructive style of coping (Mini-MAC), indicated by an F value of 15.18, an effect size (ηp2) of 0.33, and a *p*-value < 0.001, favoring the EG. The group × time interaction effect was significant for a destructive style of coping (β = −2.31, SE =0.57, *p* < 0.001) and not significant for a constructive style of coping (*p* = 0.09), suggesting that the change in scores over time differed between groups only for the destructive style of coping. The results of the LMM analysis are presented in detail in Table 3. A post hoc analysis indicated significant unfavorable differences within the CG between T0 and T2 (*p* < 0.001), between T0 and T3 (*p* < 0.001), and T1 and T2 (*p* = 0.04), as well as between groups in the T2 time point (*p* < 0.001) and T3 time point (*p* = 0.005). The mean [SD] scores were higher for the EG in the destructive style of coping at T0 (31.82 [6.38] vs. 27.31 [8.60]) and lower at T1 (28.24 [6.37] vs. 33.00 [5.34]), T2 (28.47 [2.90] vs. 40.25 [3.22]), and T3 (30.18 [2.16] vs. 38.75 [4.34]). There was no significant group × time interaction in the analysis of a constructive style of coping (*p* = 0.31), with mean [SD] scores higher in the EG at all time points (T0—43.53 [5.80] vs. 43.13 [4.60], T1—41.47 [4.39] vs. 39.13 [3.22], T2—37.53 [2.40] vs. 35.13 [2.80], and T3—38.06 [2.28] vs. 34.25 [2.93]). The style of coping assessment for both groups at the four measured time points is presented in Figure 2.

**Figure 3:**
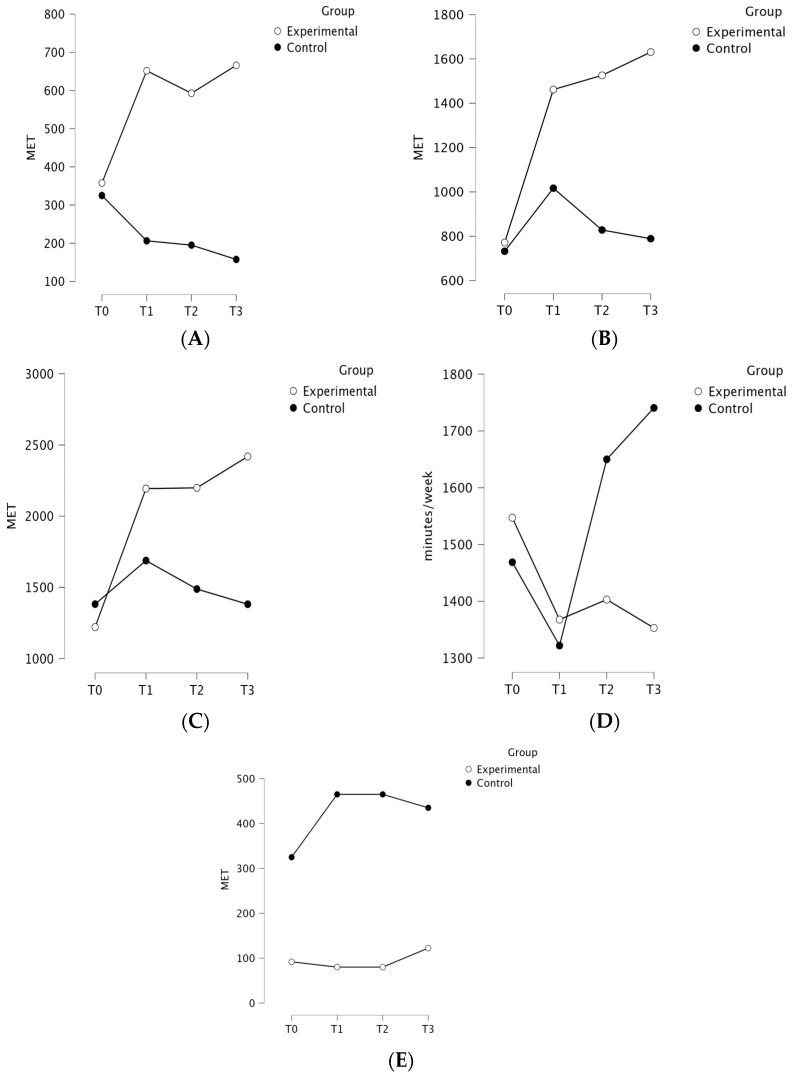
Comparison of moderate activity (**A**), walking (**B**), overall score (**C**), sedentary time (**D**), and intensive activity (**E**) (IPAQ) for the experimental and control groups at all measured time points.

The ANOVA revealed a significant group × time interaction for moderate PA (IPAQ), indicated by an F value of 6.19, an effect size (ηp2) of 0.17, and a *p*-value < 0.001, favoring EG. The mean [SD] scores were higher for the EG in moderate PA at T0 (357.65 [661.13] vs. 325.00 [443.53]), T1 (651.77 [1005.36] vs. 206.26 [267.40]), T2 (592.94 [987.14] vs. 195.00 [267.88]), and T3 (665.88 [1019.21] vs. 157.50 [210.51]). The ANOVA revealed a significant group × time interaction for walking activity (IPAQ), indicated by an F value of 2.81, an effect size (ηp2) of 0.08, and a *p*-value of 0.04, favoring the EG. The group × time interaction effect was significant for moderate activity (β = 69.31, SE = 26.31, *p* = 0.02) and overall IPAQ score (β = 75.68, SE = 75.68, *p* = 0.02) and not significant for walking (*p* = 0.07), sedentary time (*p* = 0.10), and intensive activity (*p* = 0.55), suggesting that the change in scores over time differed between groups only for moderate activity and overall IPAQ score. The results of the LMM analysis are presented in detail in Table 3. A post hoc analysis indicated significant differences within the EG between the T0 and T3 (*p* = 0.023) time points. The mean [SD] scores were higher for the EG in walking activity at T0 (771.62 [627.56] vs. 732.19 [777.96]), T1 (1461.71 [1662.32] vs. 1016.81 [966.34]), T2 (1525.77 [1714.00] vs. 828.09 [767.80]), and T3 (1630.59 [1790.42] vs. 788.91 [761.54]). The ANOVA revealed a significant group × time interaction for overall activity (IPAQ), indicated by an F value of 4.78, an effect size (ηp2) of 0.13, and a *p*-value of 0.004, favoring the EG. The post hoc analysis indicated significant differences within the EG between the T0 and T1 (*p* = 0.017), T0 and T2 (*p* = 0.016), and T0 and T3 (*p* < 0.001) time points. The mean [SD] scores were higher for the EG in overall activity at T0 (1221.03 [914.43] vs. 1382.19 [1191.93]), T1 (2193.47 [2033.66] vs. 1688.06 [1465.90]), T2 (2198.71 [2154.98] vs. 1488.09 [1280.24]), and T3 (2418.82 [2273.53] vs. 1381.41 [1240.89]). There was no significant group × time interactions in the analysis of time spent sitting (*p* = 0.27) and high-intensity activity (*p* = 0.35). The IPAQ scores for both groups at the four measured time points are presented in Figure 3.

**Figure 4:**
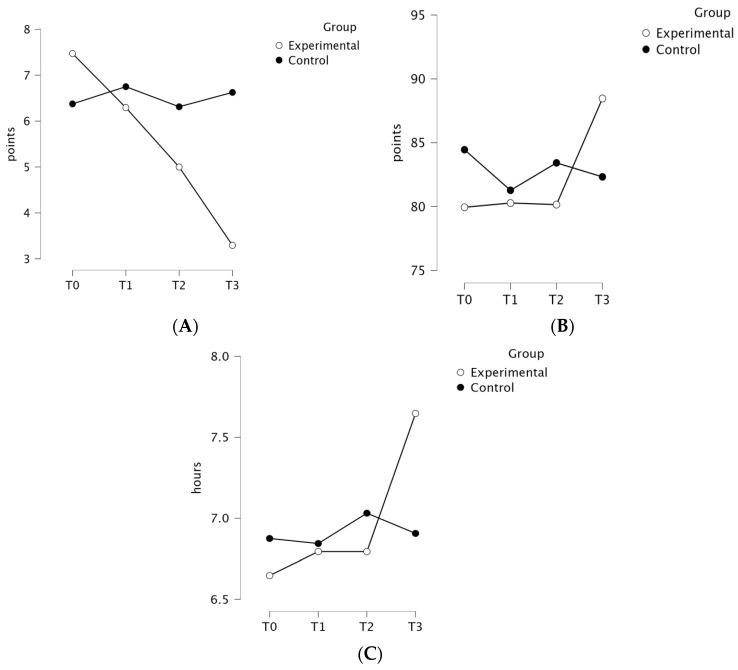
Comparison of sleep quality (**A**), sleep efficiency (**B**), and sleeping time (**C**) (PSQI) outcomes for the experimental and control groups at all measured time points.

The ANOVA revealed a significant group × time interaction for sleep quality (PSQI), indicated by an F value of 8.06, an effect size (ηp2) of 0.21, and a *p*-value < 0.001, favoring the EG. The group × time interaction effect was significant for sleep quality (β = −0.71, SE = 0.19, *p* < 0.01) and not significant for sleep efficiency (*p* = 0.12) and sleeping time (*p* = 0.20), suggesting that the change in scores over time differed between groups only for sleep quality. The results of the LMM analysis are presented in detail in Table 3. The post hoc analysis indicated significant differences within the EG between the T0 and T2 (*p* = 0.048), T0 and T3 (*p* < 0.001), and T1 and T3 (*p* = 0.005) time points. The mean [SD] scores were higher for the EG in sleep quality at T0 (7.47 [3.54] vs. 6.38 [4.06]) and lower at T1 (6.29 [3.77] vs. 6.75 [3.89]), T2 (5.00 [3.16] vs. 6.31 [3.93]), and T3 (3.29 [2.69] vs. 6.63 [3.74]). There was no significant group × time interaction in the analysis of sleep efficiency (*p* = 0.24) and sleeping time (*p* = 0.25), with a trend in sleep efficiency (3B) and sleeping time (3C) changes favoring the EG at T3 (sleep efficiency—88.46 [7.90] vs. 82.32 [6.41], sleeping time—7.65 [1.36] vs. 6.91 [0.82]). The PSQI results for both groups at the four measured time points are presented in Figure 4.

**Figure 5:**
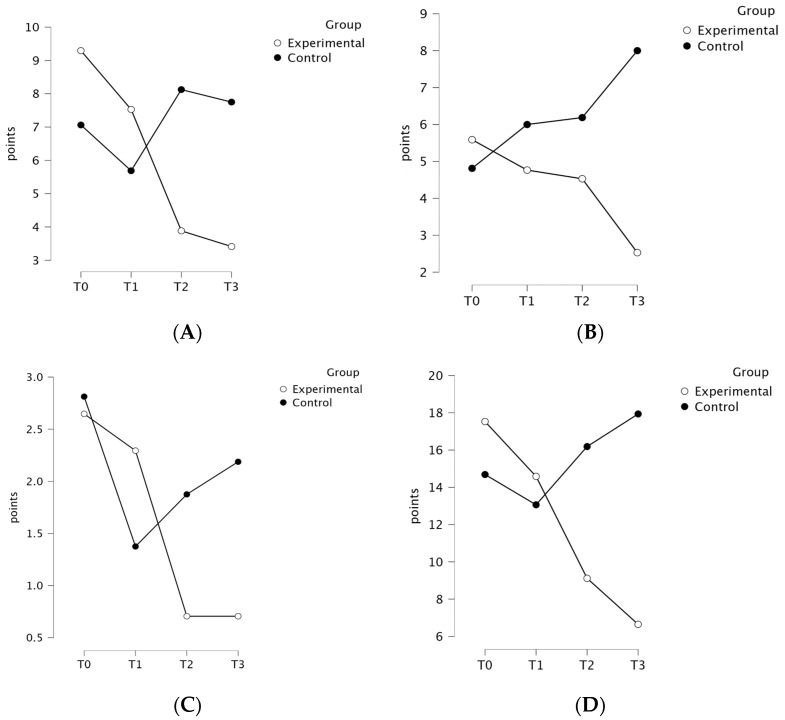
Comparison of (**A**) anxiety levels (HADS-A), (**B**) depression levels (HADS-D), (**C**) aggression levels (HADS-M), and (**D**) overall HADS scores for the experimental and control groups at all measured time points.

The ANOVA revealed a significant group × time interaction for anxiety levels (HADS-A), indicated by an F value of 9.51, an effect size (ηp2) of 0.24, and a *p*-value < 0.001, favoring the EG. The post hoc analysis indicated significant differences within the EG between the T0 and T2 (*p* = 0.006) and T0 and T3 (*p* = 0.002) time points. The mean [SD] scores were higher for the EG regarding anxiety levels at T0 (9.29 [4.48] vs. 7.06 [4.81]) and T1 (7.53 [4.77] vs. 5.69 [4.91]) and lower at T2 (3.88 [2.34] vs. 8.13 [1.89]) and T3 (3.41 [3.02] vs. 7.75 [1.77]). The ANOVA revealed a significant group × time interaction for depression levels (HADS-D), indicated by an F value of 7.13, an effect size (ηp2) of 0.19, and a *p*-value < 0.001, favoring the EG. The post hoc analysis indicated significant differences between groups at the T3 (*p* = 0.002) time point, favoring the EG. The mean [SD] scores were higher for the EG in depression levels at T0 (5.59 [3.83] vs. 4.81 [4.92]) and lower at T1 (4.77 [3.58] vs. 6.00 [ 3.29]), T2 (4.53 [2.58] vs. 6.19 [1.47]), and T3 (2.53 [2.40] vs. 8.00 [2.19]). The ANOVA revealed a significant group × time interaction for aggression levels (HADS-M), indicated by an F value of 5.16, an effect size (ηp2) of 0.14, and a *p*-value of 0.002, favoring the EG. The group × time interaction effect was significant for anxiety levels (β = −1.29, SE = 0.30, *p* < 0.001), depression levels (β = −0.96, SE = 0.24, *p* < 0.01) aggression (β = −0.31, SE = 0.11, *p* < 0.01), and overall HADS-M score (β = −2.56, SE = 0.57, *p* < 0.001), suggesting that the change in scores over time differed between groups for all HADS parameters. The results of the LMM analysis are presented in detail in Table 3. The post hoc analysis indicated significant differences within the EG between the T0 and T2 (*p* = 0.025) and T0 and T3 (*p* = 0.025) time points. The mean [SD] scores were higher for the EG in aggression levels at T1 (2.29 [2.02] vs. 1.38 [1.36]) and lower at T0 (2.65 [1.37] vs. 2.81 [1.72]), T2 (0.71 [0.77] vs. 1.88 [1.31]), and T3 (0.71 [0.85] vs. 2.19 [1.52]). The ANOVA revealed a significant group × time interaction for overall HADS scores, indicated by an F value of 9.54, an effect size (ηp2) of 0.24, and a *p*-value < 0.001, favoring the EG. The post hoc analysis indicated significant differences within the EG between the T0 and T2 (*p* = 0.046) and T0 and T3 (*p* = 0.002) time points, as well as between groups at the T3 time point (*p* = 0.004), favoring the EG. The mean [SD] scores were higher for the EG in the overall HADS scores at T0 (17.53 [7.88] vs. 14.69 [10.46]) and T1 (14.59 [8.53] vs. 13.06 [8.65]) and were lower at T2 (9.12 [4.05] vs. 16.19 [2.20]) and T3 (6.65 [5.18] vs. 17.94 [3.62]). The HADS scores for both groups at the four measured time points are presented in Figure 5.

## 4. Discussion

Due to the highly debilitating effects of oncological treatment, preparation for the healing process is extremely important. The main goals of prehabilitation are to improve the psychophysical condition and motivation for PA. Developing healthy habits in patients allows them to improve and maintain an appropriate level of emotional well-being during cancer treatment.

The last ten years have contained growing amounts of research aiming at assessing VR’s potential as a supplementary and alternative therapeutic method. These tendencies are described in a recent meta-analysis by Rutkowski et al. [20]. VR has gained popularity in scientific research; however, in the included studies, VR has been used as a distraction method rather than a treatment method. Although the use of this technology differed in its aims, it is indisputable that most available VR studies cover the aspects of mental well-being. The existing studies are insufficient to confirm the benefits of VR rehabilitation as a standalone intervention compared to standard therapy. This may be caused by small sample sizes and the poor quality of the published papers. Due to VR interventions’ high efficiency for mental treatment, we decided to investigate the technologies’ effectiveness as an independent treatment method.

The results of the performed controlled trial are in line with the results of the meta-analysis. Using VR as a prehabilitation method caused a significant reduction in depressive symptoms favoring the EG group, confirmed by the HADS results. A reduction in anxiety levels has also been confirmed by the HADS results, favoring the VR group. The analysis has shown a more destructive style of coping with the disease in the CG, compared to the VR group. The assessed PA also seemed to be on a higher level in the EG, in terms of moderate and overall activity. According to the results, patients using VR therapy had a better quality of sleep compared to the CG. Also, the aggression levels and overall HADS scores significantly favored the EG.

The results also confirm the previous outcomes from the VR TierOne device research. The same technology was used in a study involving a chronic obstructive pulmonary disease population. The investigation by Rutkowski et al. showed a significant reduction in the severity of depressive and stress symptoms [30]. In a study by Jóźwik et al. [23], similar results were obtained in a group of patients with coronary artery disease. The same intervention in elderly women with depressive symptoms by Szczepańska-Gieracha et al. [31] confirmed that the system can be effectively used in psychophysical well-being disorders, regardless of health condition and diagnosis.

The reason for the high importance of improving the psychophysical state of cancer patients during prehabilitation is mood and general well-being, which have been proven to have an impact on therapeutic effectiveness and patient participation during the healing process. Jóźwik et al. [32] confirmed that VR can improve the patient’s commitment to treatment, as well as improving the overall effects of rehabilitation. Additionally, the results show a deterioration in stress levels after VR TierOne interventions.

According to previous research, VR has the potential to be used as a tool for sleep quality improvement. A study by Horesh et al. [33] investigated the effects of VR on ovarian and BC patient well-being. The primary outcomes of this study were stress, distress, the general quality of life, quality of sleep, and illness perception. Even though the study was conducted on a small population, significant improvements were noted in participant well-being.

Another benefit resulting from the use of VR was described by Evans et al. [34]. The study conclusions suggested VR therapy can lead to an increase in PA in different populations. A study by Qian et al. [35] compared the effects of VR treatment to conventional physical training. In the systematic review, physiological, psychological, and rehabilitative outcomes were assessed during the use of VR. The VR intervention had a positive impact on all the described parameters. Unfortunately, the paper points to a research gap in the quality of empirical VR studies.

The previous results of a pilot study in a smaller sample size are in line with the above manuscript and confirm that similar interventions must be implemented as soon as possible in the treatment process [36]. Both studies include patients shortly after the diagnosis of BC. The results showed the importance of prehabilitation. Additionally, the manuscripts describe the benefits of using VR as prehabilitation treatment. This is the time that should be used not only to prepare the patient for the procedure itself and its negative effects but also to improve their already disturbed general well-being. Measurements have shown the first two weeks after diagnosis can be used to introduce effective and lasting therapeutic methods affecting the quality of sleep, the motivation for PA, and the level of anxiety and depression, as well as ways for coping with the disease.

However, against the background of the available bibliography, further, wider research on the effectiveness of VR in the improvement process seems justified, as several limitations in our study could not be avoided. It is difficult to gain insight into a patient’s overall profile, which could mask the existence of factors influencing the obtained results. We do not know the patient’s lifestyle before the diagnosis, we do not know how much of a contribution the support of family and loved ones could have had to their well-being, and whether other professional, social, or economic factors could have disturbed the results. Also, the lack of blinding the patients could be considered a limitation. However, we believe that scientific accuracy and the use of standardized measurement tools have minimized the risk of bias.

## 5. Conclusions

VR interventions seem to improve general well-being, especially in terms of depression and anxiety symptoms, improve the style of coping with the disease, motivate a higher and more intensive PA level, and improve the quality of sleep. Taking into account the possible deterioration of the patient’s psychophysical condition with the initiation of oncological treatment, future research should also include an assessment of the impact of VR depending on the treatment implemented. Also, studies on larger populations would give a more precise view of the effect of this type of intervention.

## Figures and Tables

**Table 1:** Cancer stage characteristics.

Cancer Stage *n* (%)	Experimental	Control	*p*-Value *
cTis (DCIS)	1 (6)	4 (25)	0.42
cT1N0M0	4 (23)	7 (45)	
cT1N1M0	1 (6)	0 (0)	
cT1N2M0	1 (6)	0 (0)	
cT2N0M0	2 (12)	1 (6)	
cT2N1M0	2 (12)	1 (6)	
cT2N3M0	1 (6)	0 (0)	
cT2N3M1	1 (6)	0 (0)	
cT3N0M0	3 (17)	1 (6)	
cT3N1M0	1 (6)	0 (0)	
cT3N1M1	0 (0)	1 (6)	
cT3N3M0	0 (0)	1 (6)	

Notes: *—Chi-square test.

**Table 2:** Baseline demographic characteristics.

Variable	Experimental	Control	*p*-Value *
*n*	17	16	-
Age, [years]; mean (SD)	56.68 (11.26)	59.88 (10.72)	0.41
BMI, [kg/m^2^]; mean (SD)	26.94 (6.16)	28.51 (5.89)	0.46
Education, *n* (%)			0.10
Primary/Vocational	1 (6)	5 (31)	
Secondary	7 (41)	7 (44)	
Incomplete Higher Education	0 (0)	0 (0)	
Higher Education	9 (53)	4 (25)	
Marital status, *n* (%)			0.77
Single	4 (25)	3 (18)	
Married	10 (59)	9 (56)	
Cohabiting	1 (6)	0 (0)	
Widowed	1 (6)	2 (13)	
Divorced	1 (6)	2 (13)	
Fertility, *n* (%)			0.86
0	4 (24)	3 (18)	
1	4 (24)	3 (18)	
2	6 (35)	8 (50)	
3 or more	3 (17)	2 (14)	
Material status, *n* (%)			0.06
Bad	1 (6)	0 (0)	
Average	2 (12)	8 (50)	
Good	12 (71)	8 (50)	
Very Good	2 (12)	0 (0)	

Notes: SD—standard deviation, BMI—body mass index, *—*t*-test or Chi-square test as appropriate.

**Table 3:** Results of linear mixed models analysis.

Effect	Estimate	SE	t	*p*
Constructive style of coping (Mini-MAC)				
Group	−0.15	0.92	−0.17	0.87
Time	−2.54	0.38	−6.77	<0.001
Group × Time	0.51	0.29	1.78	0.09
Destructive style of coping (Mini-MAC)				
Group	3.21	1.85	1.73	0.10
Time	1.84	0.46	4.00	<0.001
Group × Time	−2.31	0.57	−4.03	<0.001
Sleep quality (PSQI)				
Group	1.19	0.93	1.28	0.22
Time	−0.68	0.17	−3.91	<0.01
Group × Time	−0.71	0.19	−3.67	<0.01
Sleep efficiency (PSQI)				
Group	−4.15	2.77	−1.49	0.15
Time	1.02	1.12	0.91	0.37
Group × Time	1.53	0.94	1.62	0.12
Sleeping time (PSQI)				
Group	−0.30	0.33	−0.91	0.38
Time	0.16	0.11	1.48	0.16
Group × Time	0.14	0.10	1.33	0.20
HADS				
Group	4.64	2.00	2.32	0.03
Time	−1.25	0.63	−1.99	0.06
Group × Time	−2.56	0.57	−4.53	<0.001
HADS-D				
Group	1.46	0.86	1.71	0.11
Time	0.02	0.30	0.07	0.95
Group × Time	−0.96	0.24	−3.96	<0.001
HADS-A				
Group	2.66	1.10	2.42	0.03
Time	−0.84	0.28	−3.02	0.01
Group × Time	−1.29	0.30	−4.28	<0.001
HADS-M				
Group	0.52	0.34	1.54	0.14
Time	−0.44	0.14	−3.10	<0.01
Group × Time	−0.31	0.11	−2.77	<0.01
IPAQ-overall				
Group	−204.60	175.37	−1.17	0.26
Time	169.25	72.11	2.35	0.03
Group × Time	190.62	75.68	2.52	0.02
Walking activity (IPAQ)				
Group	−76.78	127.79	−0.60	0.56
Time	131.75	63.28	2.08	0.05
Group × Time	132.35	67.87	1.95	0.07
Moderate activity (IPAQ)				
Group	−0.26	109.43	−0.01	0.99
Time	17.28	25.52	0.68	0.51
Group × Time	69.31	26.31	2.64	0.02
Intense activity (IPAQ)				
Group	−131.06	76.36	−1.72	0.11
Time	21.79	22.46	0.97	0.35
Group × Time	−12.62	20.63	−0.61	0.55
Sedentary (IPAQ)				
Group	147.69	198.14	0.75	0.47
Time	28.89	57.54	0.50	0.62
Group × Time	−83.60	48.04	−1.74	0.10

Notes: SE—standard error, Mini-MAC—Mental Adjustment to Cancer Scale, PSQI—Pittsburgh Sleep Quality Index, HADS—Hospital Anxiety and Depression Scale (D—depression, A—anxiety), IPAQ—International Physical Activity Questionnaire.

## Data Availability

Data are available from the corresponding author upon reasonable request.

## References

[1] Anders C., Johnson R., Litton J., Phillips M., Bleyer A. (2009). Breast cancer before age 40 years. Semin. Oncol..

[2] Nakatani Y., Iwamitsu Y., Kuranami M., Okazaki S., Yamamoto K., Watanabe M., Miyaoka H. (2012). Emotional Suppression and Psychological Responses to a Diagnosis of Breast Cancer. Shinrigaku Kenkyu.

[3] Wang H.H., Chung U.L. (2012). Healthy lifestyle changes during the period before and after cancer diagnosis among breast cancer survivors. Asian Pac. J. Cancer Prev..

[4] Mehnert A., Koch U. (2007). Prevalence of acute and post-traumatic stress disorder and comorbid mental disorders in breast cancer patients during primary cancer care: A prospective study. Psychooncology.

[5] Loeffler S., Poehlmann K., Hornemann B. (2018). Finding meaning in suffering?—Meaning making and psychological adjustment over the course of a breast cancer disease. Eur. J. Cancer Care.

[6] Little M., Sayers E.J. (2004). While there’s life …: Hope and the experience of cancer. Soc. Sci. Med..

[7] Khan F., Amatya B., Pallant J.F., Rajapaksa I. (2012). Factors associated with long-term functional outcomes and psychological sequelae in women after breast cancer. Breast.

[8] Mehnert A., Hartung T.J., Friedrich M., Vehling S., Brähler E., Härter M., Faller H. (2018). One in two cancer patients is significantly distressed: Prevalence and indicators of distress. Psychooncology.

[9] Czech O.J., Matkowski R., Skórniak J., Malicka I. (2024). Psychological and Physical Well-Being in Women Diagnosed with Breast Cancer: A Comprehensive Study of Anxiety, Depression, Sleep Quality, Physical Activity, and Sociodemographic Factors. Med. Sci. Monit..

[10] Bray F., Ferlay J., Soerjomataram I., Siegel R.L., Torre L.A., Jemal A. (2018). Global cancer statistics 2018: GLOBOCAN estimates of incidence and mortality worldwide for 36 cancers in 185 countries. CA Cancer J. Clin..

[11] Epstein N. (2014). Multidisciplinary in-hospital teams improve patient outcomes: A review. Surg. Neurol. Int..

[12] Loef M., Paepke D., Walach H. (2023). Quality of Life in Breast Cancer Patients Treated With Mistletoe Extracts: A Systematic Review and Meta-Analysis. Integr. Cancer Ther..

[13] Saevarsdottir S., Gudmundsdottir S. (2023). Mobile Apps and Quality of Life in Patients With Breast Cancer and Survivors: Systematic Literature Review. J. Med. Internet Res..

[14] Javan Biparva A., Raoofi S., Rafiei S., Masoumi M., Doustmehraban M., Bagheribayati F., Ghashghaee A. (2023). Global depression in breast cancer patients: Systematic review and meta-analysis. PLoS ONE.

[15] Krok D., Telka E., Moroń M. (2023). Marital satisfaction, partner communication, and illness acceptance among couples coping with breast cancer: A dyadic approach. Psychooncology.

[16] Sun M., Liu C., Lu Y., Zhu F., Li H., Lu Q. (2023). Effects of Physical Activity on Quality of Life, Anxiety and Depression in Breast Cancer Survivors: A Systematic Review and Meta-analysis. Asian Nurs. Res..

[17] Morina N., Ijntema H., Meyerbroker K., Emmelkamp P.M. (2015). Can virtual reality exposure therapy gains be generalized to real-life? A meta-analysis of studies applying behavioral assessments. Behav. Res. Ther..

[18] Beidel D.C., Frueh B.C., Neer S.M., Lejuez C.W. (2017). The efficacy of trauma management therapy: A controlled pilot investigation of a three-week intensive outpatient program for combat-related PTSD. J. Anxiety Disord..

[19] Falconer C.J., Rovira A., King J.A., Gilbert P., Antley A., Fearon P., Brewin C.R. (2016). Embodying self-compassion within virtual reality and its effects on patients with depression. BJPsych Open.

[20] Rutkowski S., Czech O., Wrzeciono A., Kiper P., Szczepanska-Gieracha J., Malicka I. (2021). Virtual reality as a chemotherapy support in treatment of anxiety and fatigue in patients with cancer: A systematic review and meta-analysis and future research directions. Complement. Ther. Med..

[21] Chirico A., Lucidi F., De Laurentiis M., Milanese C., Napoli A., Giordano A. (2016). Virtual reality in health system: Beyond entertainment. A mini-review on the efficacy of VR during cancer treatment. J. Cell Physiol..

[22] Hoffman H.G., Chambers G.T., Meyer W.J., Arceneaux L.L., Russell W.J., Seibel E.J., Patterson D.R. (2011). Virtual reality as an adjunctive non-pharmacologic analgesic for acute burn pain during medical procedures. Ann. Behav. Med..

[23] Jóźwik S., Cieślik B., Gajda R., Szczepańska-Gieracha J. (2021). Evaluation of the Impact of Virtual Reality-Enhanced Cardiac Rehabilitation on Depressive and Anxiety Symptoms in Patients with Coronary Artery Disease: A Randomised Controlled Trial. J. Clin. Med..

[24] Mazurek J., Kiper P., Cieślik B., Rutkowski S., Mehlich K., Turolla A., Szczepańska-Gieracha J. (2019). Virtual reality in medicine: A brief overview and future research directions. Hum. Mov..

[25] Kiper P., Przysiężna E., Cieślik B., Broniec-Siekaniec K., Kucińska A., Szczygieł J., Turek K., Gajda R., Szczepańska-Gieracha J. (2022). Effects of Immersive Virtual Therapy as a Method Supporting Recovery of Depressive Symptoms in Post-Stroke Rehabilitation: Randomized Controlled Trial. Clin. Interv. Aging..

[26] Watson M., Greer S., Young J., Inayat Q., Burgess C., Robertson B. (1988). Development of a questionnaire measure of adjustment to cancer: The MAC scale. Psychol. Med..

[27] Hagströmer M., Oja P., Sjöström M. (2006). The International Physical Activity Questionnaire (IPAQ): A study of concurrent and construct validity. Public Health Nutr..

[28] Casement M.D., Harrington K.M., Miller M.W., Resick P.A. (2012). Associations between Pittsburgh Sleep Quality Index factors and health outcomes in women with post-traumatic stress disorder. Sleep Med..

[29] Zigmond A.S., Snaith R.P. (1983). The hospital anxiety and depression scale. Acta Psychiatr. Scand..

[30] Rutkowski S., Szczegielniak J., Szczepańska-Gieracha J. (2021). Evaluation of the Efficacy of Immersive Virtual Reality Therapy as a Method Supporting Pulmonary Rehabilitation: A Randomized Controlled Trial. J. Clin. Med..

[31] Szczepańska-Gieracha J., Cieślik B., Serweta A., Klajs K. (2021). Virtual Therapeutic Garden: A Promising Method Supporting the Treatment of Depressive Symptoms in Late-Life: A Randomized Pilot Study. J. Clin. Med..

[32] Jóźwik S., Cieślik B., Gajda R., Szczepańska-Gieracha J. (2021). The Use of Virtual Therapy in Cardiac Rehabilitation of Female Patients with Heart Disease. Medicina.

[33] Horesh D., Kohavi S., Shilony-Nalaboff L., Rudich N., Greenman D., Feuerstein J.S., Abbasi M.R. (2022). Virtual Reality Combined with Artificial Intelligence (VR-AI) Reduces Hot Flashes and Improves Psychological Well-Being in Women with Breast and Ovarian Cancer: A Pilot Study. Healthcare.

[34] Evans E., Naugle K.E., Kaleth A.S., Arnold B., Naugle K.M. (2021). Physical Activity Intensity, Perceived Exertion, and Enjoyment During Head-Mounted Display Virtual Reality Games. Games Health J..

[35] Qian J., McDonough D.J., Gao Z. (2020). The Effectiveness of Virtual Reality Exercise on Individual’s Physiological, Psychological and Rehabilitative Outcomes: A Systematic Review. Int. J. Environ. Res. Public Health.

[36] Czech O., Siewierska K., Krzywińska A., Skórniak J., Maciejczyk A., Matkowski R., Szczepańska-Gieracha J., Malicka I. (2022). Virtual Therapy Complementary Prehabilitation of Women Diagnosed with Breast Cancer-A Pilot Study. Int. J. Environ. Res. Public Health.

